# Exploring the expression of depression and distress in aboriginal men in central Australia: a qualitative study

**DOI:** 10.1186/1471-244X-12-97

**Published:** 2012-08-01

**Authors:** Alex Brown, Ushma Scales, Warwick Beever, Bernadette Rickards, Kevin Rowley, Kerin O’Dea

**Affiliations:** 1 Baker IDI Heart and Diabetes Institute, W and E Rubuntja Building, Alice Springs Hospital, Gap Road, Alice Springs, NT, 0870, Australia; 2 Central Australian Aboriginal Congress, 25 Gap Road, Alice Springs, NT, 0870, Australia; 3 Onemda VicHealth Koori Health Unit and Centre for Health & Society, School of Population Health, Level 4, 207 Bouverie Street, The University of Melbourne, Melbourne, VIC, 3010, Australia; 4 Sansom Institute for Health Research, University of South Australia, GPO Box 2471, Adelaide, South Australia, 5001, Australia

## Abstract

**Background:**

Despite being at heightened risk of developing mental illness, there has been little research into the experience of depression in Australian Aboriginal populations. This study aimed to outline the expression, experience, manifestations and consequences of emotional distress and depression in Aboriginal men in central Australia.

**Methods:**

Utilizing a grounded theory approach, in depth semi-structured interviews were conducted with 22 theoretically sampled young, middle aged and senior Aboriginal men and traditional healers. Analysis was conducted by a single investigator using constant comparison methods.

**Results:**

Depressive symptoms were common and identifiable, and largely consistent with symptom profiles seen in non-Aboriginal groups. For Aboriginal men, depression was expressed and understood as primarily related to weakness or injury of the spirit, with a lack of reference to hopelessness and specific somatic complaints. The primary contributors to depression related to the loss of connection to social and cultural features of Aboriginal life, cumulative stress and marginalisation.

**Conclusions:**

Depression and depressive symptomatology clearly exists in Aboriginal men, however its determinants and expression differ from mainstream populations. Emotions were understood within the construction of spirit, *Kurunpa*, which was vulnerable to repetitive and powerful negative social forces, loss, and stress across the life course, and served to frame the physical and emotional experience and expression of depression.

## Background

Growing international interest has focused attention on the need to overcome health disadvantage experienced by the world’s more than 370 million Indigenous peoples [1]. Among the long and seemingly intractable list of determinants, mental health and social and emotional wellbeing loom as critical priorities.

Disparities in health outcomes experienced by Indigenous Australians are as large as those seen in any other high-income country. Life expectancy differentials are between 10 and 20 years [2,3]. Indigenous males experience a disproportionate burden of ill health. They are more likely to die from almost any cause and at any age than are non-indigenous males, have the lowest life expectancy, and high rates of substance misuse, suicide, and incarceration [4]. The reasons for these inequalities are incompletely understood, but mental illness is considered to be key contributor [5]. Racism, family disconnectedness, community dysfunction, and social disadvantage constitute chronic stressors among Aboriginal communities, and have ongoing effects on the mental health and wellbeing of individuals [6,7]. The loss of land, culture and identity, covert and overt racism, marginalisation and powerlessness have been highlighted by Indigenous peoples as fundamental causes of ill health [1,7-10].

Despite international interest in mental illness among American Indian [11-13] and non-European ethnic groups [14-17], there has been surprisingly little empirical research exploring the experience, manifestations and consequences of depression among Aboriginal people within Australia. There are several contributors to the lack of progress. Aboriginal and Torres Strait Islander communities exhibit significant heterogeneity in their social, demographic, geographical, cultural, linguistic and historical experiences. Seeking any such ‘pan-Aboriginal’ reality to the experience of psychopathology is fraught with practical, social, ethical, and methodological challenges. The concept of depression is also expressed linguistically in varying ways in different cultural groups [18], particularly as it relates to the expression of symptoms, and the nature, antecedents and consequences of depression as an illness [19]. Culture can impact on the way in which an individual manifests symptoms of mental illness [13]. Culture may determine or frame causative, precipitating or maintenance factors, influencing the onset, symptom profile, impact, course and outcome of mental illness [13,17].

Despite the pan-human capacity for sadness and grief, this does not, by extension, mean that depression, as a construct, is universal [20]. That being said, however, depression has been identified in almost all populations for which it has been sought, including Aboriginal and Torres Strait Islander people within Australia [21-24]. Importantly, differences have been demonstrated across cultures in the experience, expression and consequences of a range of emotional terms and illnesses [19,21,24,25]. It is therefore essential to understand the broader social, political, historical, physical, spiritual and psychological worlds in which health and conversely, illness, is constructed and experienced to better understand the health and social disadvantage of Aboriginal people.

The Men, Hearts and Minds Study was established as a multi-stage mixed methods project to explore the interplay of psychosocial factors and cardiovascular risk in Aboriginal people.

The aim of the qualitative component of the study reported here was to explore and ultimately outline the expression, experience, manifestations and consequences of emotional distress and depression in Aboriginal men in Central Australia specifically to enhance the development of methods that could accurately measure depressive symptoms in the target population. It was based on the underlying premise that *depression and depressive symptoms are a universal experience, bound to common biological features and theoretical domains of experience and psychology, but that local idioms of distress, expressions and modes of communication, would be culturally specific*.

## Methods

"*What scientists hold stock in is only what they can measure. But you can’t measure the mind or spirit. You can’t weigh it, you can’t deconstruct it. But only if we do will they see that Aboriginal people are spectators to the death of their culture, their lives…We watch as our culture dies…How are you going to measure that?*"

"Senior Aboriginal man, Alice Springs."

In order to avoid assumptions of cross cultural equivalence and pre-imposed ideas of the conceptualization of depression, qualitative methods informed by both ethnographic and grounded theory approaches were employed to explore and theorise the way in which depression and emotional distress are expressed, experienced and understood by Aboriginal men themselves [26,27].

### Sampling

The study utilized a purposive sampling strategy aimed firstly at identifying and recruiting *Ngangkari Tjuta* (or traditional healers) who would participate in detailed interviews which we anticipated would provide a full understanding of the expression of depression in order to examine meanings, interpretations, processes and theory [28]. *Ngangkari Tjuta* maintain a critical place within the fabric of contemporary Aboriginal life in Central Australia [29], as essential protectors of the community’s wellbeing at times of trouble, sickness, and sadness.

Through undertaking constant comparison of data, via progressive inductive analysis of the interviews [30] conducted with *Ngangkari*, it became clear that there was an imperative to augment the sample with senior knowledgeable men, younger men, and men in other settings. Critically, in terms of conducting research within Aboriginal communities and settings, theoretical sampling [28] was verified through the traditional healers themselves who instructed the researchers towards sampling further amongst young [20-35 yrs], middle-aged [35-55 yrs] and senior men [over 55 yrs], and men from different language groups. Finally, we approached several key community informants who had lived within traditional Aboriginal community and cultural life, as well as within mainstream Australian society. Consistent with the concurrent data collection, coding and analysis that underpins the grounded theory approach, recruitment continued until thematic saturation was obtained.

### Context/Settings

Data were collected over a 6-month period in 2006, involving the conduct of 17 interviews across seven remote communities, and five interviews conducted within Alice Springs in the Northern Territory of Australia. In total, seven were conducted with *Ngangkari Tjuta*; six were conducted with senior Aboriginal men (5 remote community members; 1 urban resident); three were conducted with middle-aged Aboriginal men (1 remote; 2 urban); and six with young Aboriginal men (4 remote, 2 urban). The vast majority of interviews were conducted in community settings, at a place deemed appropriate by the participants themselves. The interviews usually lasted two to three hours, often requiring significant ‘work’ to make the space separate from the communities’ activities of daily life and safe, in so far as the discussion may have touched on gender sensitive issues, requiring the maintenance of confidentiality and creation of a ‘male only space’[10]. Often men chose sites which had particular cultural significance to them, in order to relay the importance of the content and context of their narratives.

### Procedures

An important component of the methodology included the use of an introductory story or fictional vignette Additional file 1: Appendix 1, developed by the CI [AB] and the study’s senior anthropologist [US] to contextualise and focus the discussion generated within interviews around the experience of mental illness and distress. Vignettes have been used in research contexts for over 25 years [31] and usually take the form of short stories about hypothetical characters in specified circumstances, to whose situation the interviewee is invited to respond. Our vignette focused on an Aboriginal man within a remote community who had recently experienced emotional and somatic complaints prior to attendance at a remote community health centre. The vignette outlined symptoms reflective of a major depressive episode as defined within existing diagnostic criteria [32].

The vignette was followed by a semi-structured interview Additional file 2: Appendix 2, which was based on modified open and close ended questions utilized within cross-cultural psychiatric research among Hopi Indians [11], Inuit [25], Australian Aboriginal populations [21,33], and further guided by questions for exploring the cultural context of depressive illness proposed by Marsella [20] and others [34]. Finally, a list of depressive symptoms collated from the principal domains of mood disorders within DSM-IV [32] and iteratively augmented with identified symptom profiles emerging from interviews as the study progressed was tabulated Additional file 3: Appendix 3. Each participant was asked to endorse the existence (or absence) of each specific symptom. This served both as a means of summarising the findings of the interviews and seeking consensus across participants.

Each interview was performed by two experienced interviewers, namely the CI [AB] and either a senior anthropologist [US] or research officer [WB], in the language of choice of the participant following informed consent. Interviews conducted within remote communities often involved many ‘observers’, enlisted by the participants to ‘witness’ their accounts, and as a form of validation of the story proffered. Most interviews were digitally recorded then transcribed, the remainder involving the documentation of extensive field notes taken by interviewers.

### Analysis

All analyses were conducted by a single investigator [AB] and commenced with the generation of a reflective interview summary which was collated as soon as possible after the interview was completed. This comprised preliminary open coding, or the allocation of codes to key concepts emerging within interview content [28], as well as a description of the discussion, including non-verbal cues, and the setting of the interview. Identified codes were then compared in an ongoing fashion in subsequent interviews so that data collection and analysis continued simultaneously, consistent with a grounded theory approach [35,36]. Key emergent themes derived from the coding process were then broadly conceptualised, defined and discussed with several key informants, and interconnections between themes explored. These discussions involved the study’s senior anthropologist, a bi-lingual researcher and community advisor, an Aboriginal female researcher and health practitioner, an Aboriginal elder from a remote community and an Aboriginal elder from Alice Springs. They occurred over a period of twelve months, and involved extensive reshaping of concept, meaning and coverage of the major thematic areas of interest identified in interviews.

Ethics approval was received from the Central Australian Human Research Ethics Committee.

## Results

The research team was not prepared for the immediacy of entry into participants' emotional lives. The fictional narrative, intended as an introduction to the objectives, parameters and concepts the study sought to explore, was instead understood as a ‘real story’, an accurate reflection of the experiences of many Aboriginal men. In this sense, the fictional narrative unwittingly served to link a story of ‘some other man’ to the very personal, broader and concrete narratives of distress and emotional experiences of participants. Most men, when asked to recall if they had ever witnessed or known of someone who had similar concerns and symptoms to those outlined in the vignette, related directly to their own stories of emotional distress.

"*There are plenty of Anangu [Aboriginal] men like this one [Kunmanara’s Story], like me, I feel like that. Thinking about all the people around me…worry, worry, worry…Now I look around me, I see my friends, old mates, even young blokes have finished up [died] early. This is a big cause of sadness…I feel those things too [Kunmanara’s Story].*"

"Senior Aboriginal man, remote community."

Depressive symptoms and behaviours were extremely common amongst the participants, with all men having previously experienced significant negative emotional symptoms, if not frank distress. Depressive symptoms were seen in Aboriginal men across remote and urban settings, and across all age groups.

"*Yes…I’ve looked after my brother. He suffers from depression and all that. He’s pretty bad way. I think I suffer from it too…I do have it, you know, and that’s what a lot of people say…even my missus would say “are you depressed?”*"

"Aboriginal Health Worker, remote community"

### Recognising depression

Despite the commonness of depressive symptoms, depression was not a word or term that was frequently used, or felt by the participants to be widely understood among community men.

"*Cause you can’t put a word on it for Aboriginal people… We understand stress. But depression…it’s a word that’s not…even in our vocabulary.*"

"Young Aboriginal man, Alice Springs."

There was very little recognition of depression either as a medical diagnosis or as a cluster of negative emotional symptoms, even when informants themselves had faced significant social and emotional difficulties. For many participants, the interviews served as a catalyst for recognising previous emotional turmoil.

"*I’ve never been clinically diagnosed [with depression] but there’s been three or four times when I’ve held guns to my head, put a gun in my mouth and that kind of stuff…*"

"Young Aboriginal man, Alice Springs."

### Masking symptoms

One of the principal barriers to recognising depressive symptoms in Aboriginal men was their hesitancy to talk about their emotions. None had previously openly discussed the worries or the sadness they had experienced.

"*People don’t talk about these things [feelings]. They hold it in. The hurting…we keep to ourselves…*"

"Young Aboriginal man, remote community."

One participant spoke of the impact of forced separation and recurrent institutionalisation on his ability to openly express his feelings.

"*It costs you…growing up in institutions, missions…you never went to funerals and when you come out and you go to a funeral of a family member you don’t know how to grieve, you don’t know how to cry, you don’t know how to feel…I didn’t cry at my father’s funeral, I couldn’t cry. I didn’t know how to cry…*"

"Senior Aboriginal man, Alice Springs."

### Violence and building anger

Anger and impulsive acts of violence were prominent features of depression, particularly in the context of continual efforts to suppress the anger men felt about what had occurred in their lives.

"*Some [men] get cranky when they feel like this [depressed]. They get angry at small things, silly things. But it is the thing that makes us release all that worry and anger. Too much worry can make you explode. You keep it down, push it inside, hold on until there is too much pressure and you explode…Thinking, thinking, building up this anger until they explode. It happened to me. It built up, I hold it in, don’t let it out.*"

"Young Aboriginal man, remote community."

The primary contributors to anger were frustration, pervasive stress and worry, and the marginalisation of Aboriginal men within contemporary society. The consequence was cumulative strain on the body and mind of Aboriginal men.

"*I’m still angry now. I’ve been angry most of my life and this is the thing that is burning me out is that as I said earlier, I’m more prone to fight than to flight, but at the same time the wear and tear on my body in regards to the amount of adrenaline in your system has never switched off and it just burns you…it burns you, you get angrier and angrier.*"

"Senior Aboriginal man, Alice Springs."

### Substance misuse and depressive symptoms

Anger within the context of depression among Aboriginal men was most frequently expressed as self-destructive behaviours. Of prominence was the strong interaction of harmful levels of substance misuse with depressive symptoms. The misuse of alcohol and marijuana was considered as an attempt by men to hide their emotional distress.

"*Young fella’s use ukiri [lit. ‘green grass’; marijuana] to push down their worry, they try to drown it. They are putting on a mask. They are trying to hide, deny their feelings, but it doesn’t. They forget for a while but they never heal. They wake up the next day worse. They are building and building till they explode. I have seen this.*"

"Senior Aboriginal man, remote community*.*"

### ‘Worry’ and ‘sadness’ among Aboriginal men

‘Worry’ and ‘sadness’ were seen as the primary contributors to emotional distress, and the fundamental expression of depressed mood. Depression, as a disease state, as a lay construct, and as a symptom profile was identified by participants as being related to Aboriginal expressions of great sadness [*tjituru-tjituru*] and excessive, intrusive and repetitive worry, ‘too much thinking’, ‘too much worry’ [*kulini-kulini*].

"*Everybody has worries. Everybody has worries. Everyone has stresses in their life it’s just how you deal with them…people tend to live with those worries and they infest in them and they just get bigger and deeper and they’re just constantly weigh on the mind.*"

"Young Aboriginal man, Alice Springs."

In particular, grief and loss were important contributors to depressive symptoms.

"*It’s not known in the other half of society of the amount of funerals[Aboriginal] people go to, it’s not known…your week is just full of grief and loss. I think that’s another thing that drives people into deep, deep alcoholism, deep depression, deep suicide you know it’s…it’s sad – never-ending.*"

"Senior Aboriginal man, Alice Springs."

Depression was also seen as a consequence of recurrent, cumulative stress, disadvantage, marginalisation, oppression, forced separation and an overwhelming sense of loss.

"*Depression is a continuum in Aboriginal people’s lives, it’s the struggle from birth to death, and it’s a struggle for ever and a day just getting the recognition that you’re a human being…It really is the shadow that follows you, it’s forever there. I think the whole system of depression is come about from the need for white society to render you powerless, so that you are no longer in opposition…*"

"Senior Aboriginal man, Alice Springs."

Worry was particularly bound to factors of critical importance to the expression and survival of Aboriginal culture, expressions of self and ways of life. These were covered under the specific domains of *Tjukurpa* [the Law, or Dreaming], *Walytja* [Family]*, Ngurra* [Land], *and Kanyini* [connectedness].

Celebration and re-invigoration of cultural practice remains a pivotal component of the identity and life of Central Australian Aboriginal men. However, the dynamic and pernicious impact of colonisation, particularly its influence on Aboriginal men’s way of life, had significant negative consequences on their emotional wellbeing.

"*In the old days…there was probably no depression prior to White man’s influence. Everything was set in place, everyone knew their place, everyone knew what had to be done, everyone worked together, and everyone contributed. There were rules that weren’t broken. Everything was spot on. Whereas now there are two lots of rules, no respect, hardly any culture, everything is just confusion…*"

"Aboriginal Health Worker, remote community."

### Family and emotional distress

Family emerged as a significant contributor to emotional distress, largely as a consequence of recurrent worry for family, and grief as a result of family deaths which weighed heavily on participants.

"*Loss of family, we sad and worried…that feeling we keep for a long time. People think all the time. If I lose him, my best good friend, I worry. I look at people around me, worry for a long time. See friends together; think back about the friends lost. The grief…he thinks…then he gets sick inside.*"

"Senior Aboriginal man, remote community."

Disconnection from family was also a primary cause of worry and was seen as a direct contributor to emotional distress.

"*Someone will worry when their relatives go away and they’ll go to sleep at night and just worry and fret about them all night long…Then they’ll get sad…Kurunpa tjituru tjituru* [‘my spirit is very sad’]. *The kurunpa* [spirit] *gets weaker. When the people finally come back they’ll be OK again. This is how we are.*"

"Senior Aboriginal man, remote community."

For Aboriginal men, the close connections to family, country and all things within the sphere of their existence are incorporated within the concept of *walytja* [family]. These connections form one of the most important foundations of Aboriginal life [37]. This concept of interconnectedness, responsibility and duty to care for all things, was understood as *Kanyini* [‘To have, to hold, to care’] [38] and the maintenance of positive connections between people and place was central to well-being in Aboriginal men. As a consequence, when people were separated, or disconnected from family life, this triggered a longing for reconnection. If unable to reestablish family and community bonds, or if the separation was prolonged, participants experienced recurrent thoughts and worries for family and feelings of great sadness. This feeling of disconnection was a primary symptom of depression. In the Pitjantjatjara language, this emotion was termed *Watjilpa*.

"*So we are trying to work these words* [emotional terms for depressive symptoms] *out now. Watjilpa is one of those words. One is thinking, ‘Oh, I have to go back, ‘I’ve gone away but I’ve got to return.’ He is homesick and he is thinking…of going back to where he came from, so he can be happy… I am homesick, watjilpa.*"

"Senior Aboriginal man, remote community [Translated]."

"*Watjilpa weakens the Kurunpa [spirit].*"

"Senior Ngangkari, remote community [Translated]."

### Conceptualising depression: worry, sadness and its impact on kurunpa [spirit]

"*We need Kurunpa to be at one. The Kurunpa is like spirit, body, mind and soul of the body – when it goes you’re just meat and bones. When the Kurunpa leaves that person, the thing that drives that person to live has gone.*"

"Aboriginal man, remote community."

Whilst there was initial difficulty in accepting the use of the term ‘depression’, after careful negotiation between participants and the research team, involving discussion and explanation through the use of an introductory vignette, depression was understood. This was particularly the case for *Ngangkari Tjuta*, who frequently saw and treated people who could be described as suffering from depressive symptoms. In essence, depression was ‘visible’ to *Ngangkari Tjuta*, because it was associated with alterations, movements or weakness in someone’s spirit [*Kurunpa*].

"*We are always asked to look after these ones. There are a lot of people like Kunmanara, their kurunpa is troubled. When your kata [head] is all level and the milkali [blood] is flowing around nicely, you’re happy, pukulpa, your Kurunpa is happy and level and balanced and everything is fine…When you are worried, your kurunpa becomes tjukaru [crooked/bent] from all the worry…When it gets bent, you can’t think straight…We can see their kurunpa when it’s all crooked.*"

"Senior Ngangkari, remote community [Translated]."

The importance of *kurunpa*, and the way in which it is visualised, diagnosed, manipulated, and managed by *Ngangkari*, was central to Aboriginal men’s understanding and experience of depression, particularly in relation to the links between physical illness and emotional distress.

"*I can look into somebody and see their kurunpa. Ngangkari can see kurunpa and see sickness. I know these things. It is actually basic stuff to us really! [laughs].*"

"Senior Ngangkari, remote community [translated]."

Further, the state, strength and vitality of a persons’ *kurunpa* was central to the symptom profile of individuals experiencing depressive illness. *Kurunpa* was clearly linked to both the health of the body and of the mind, and any perceived imbalance was the primary physical and metaphysical referent for illness.

"*There are three big signs of depression in Anangu [Aboriginal people]. Mirpanpa [Anger; literally ‘hot in throat’]; tjituru tjituru pulka [great sadness] and watjilpa [homesickness]. But it’s not just feeling sadness…their spirit is sad. They think too much, kulini kulini kulini [lit. thinking, thinking, thinking]. This story, Kunmanara, a slipped kurunpa can cause all of them. People aren’t hungry, can’t hunt, sit down by themselves, they are watjilpa, not looking after family. I look inside them like X-ray and I see their kurunpa is not level.*"

"Senior Ngangkari, remote community."

## Discussion

From a descriptive stance, the most consistently expressed mood symptom among Aboriginal men experiencing depression was excessive sadness. Irritability was commonly experienced, and although the terms ‘depressed’ or ‘feeling depressed’ were understood by the majority of participants, they were used directly to explain subjective feeling by very few respondents. There was a complete absence of reference to hopelessness among participants from remote communities, and infrequent endorsement of the term as a possible expression of depression within urban dwelling participants. There was infrequent mention of somatic complaints specifically associated with depression, but abnormal sleeping (particularly early morning wakening), tiredness and gastrointestinal upset were discussed. The most commonly endorsed cognitive element of depression in Aboriginal men was the recurring and intrusive nature of excessive ‘worry’, in particular, recurrent thinking about the things that caused great sadness and concern. Suicidality was frequently endorsed as a subjective symptom of depression, and as a visible expression of depressive symptoms in others, and was recognised by the majority of participants as such. Guilt and self-reproach were virtually non-existent.

Previous research, such as Sheldon’s clinical reports of depression in Central Australian communities [21] has identified the presence of severe depressive illness in remote Aboriginal people. Further Sheldon’s reports were the first to articulate the dependability of a ‘weakened spirit’ as an indicator of depressed mood.

Existing literature has also demonstrated the existence of depression in Aboriginal communities in the Top End of Australia [39]. Cawte’s extensively documented field research [23,33], resulted in the characterization of culturally-specific classifications of depressive illness in remote Aboriginal communities. Consistent with Cawte’s work, Aboriginal participants in our study discussed the frequency of hidden or masked depressive symptoms, undifferentiated somatic complaints, lethargy and ‘vital exhaustion’, and the strong interplay between depressed mood and anger, impulsive acts of violence and substance misuse.

In this group of men, depressive symptoms were common and identifiable, and largely consistent with those seen in non-Indigenous patient groups, particularly at the severe end of the illness spectrum. Co-morbidity of depression with substance misuse was identified, as was the importance and frequency of anger in young men. The contributory impact of continual grief and loss outlined by Cawte [23] was also replicated in this study, adding weight to the proposed conceptualization of the social origins of depression in Aboriginal communities, particularly among individuals experiencing profound socio-cultural changes in the face of an increasingly dominant western culture.

In our study, worry was the most recognisable element of depression and was considered both a contributor to, and prominent element in, the construction of depression. Most notable was the consistency, repetitiveness and intrusive nature of worry. The emotional consequences were profound sadness. Worry and sadness were contributors to and consequences of each other, entering Aboriginal men into cycles of rumination, with emotional and physical illness the outcome.

There is a growing literature indicating the importance of worry and rumination in affective states. Whilst diverging views on the construction of rumination abound, it represents persistent, cyclical often depressive thinking [40], and it has been documented as an important cognitive element of affective disorders [41-44]. ‘Worry’ and rumination are closely related, but are likely to be distinct phenomena [40,45]. This distinction is important because worry is a key feature of anxiety disorders, where thoughts tend to be predominated by fear and anticipated threat, whereas depressive rumination tends to focus on past loss, grief and self-perceived failure [46]. The content of ‘worry’ for Aboriginal men in this sample was predominated by loss, grief, sadness, and mourning for the changes in their lives that had wrought social and cultural chaos. As such, the contributors to, and content and impact of ‘worry’ in this sample of Aboriginal men may serve to differentiate anxiety related worry and depressive rumination. Studies of emotion and mental illness have also highlighted the central importance of ‘worry’ and ‘too much thinking’ in the construction of depression in distinct cultural groups, including the Shona in Zimbabwe [47] and Nicaraguan women [48].

### Building a theory of depression in Aboriginal men in Central Australia

**Figure 1:**
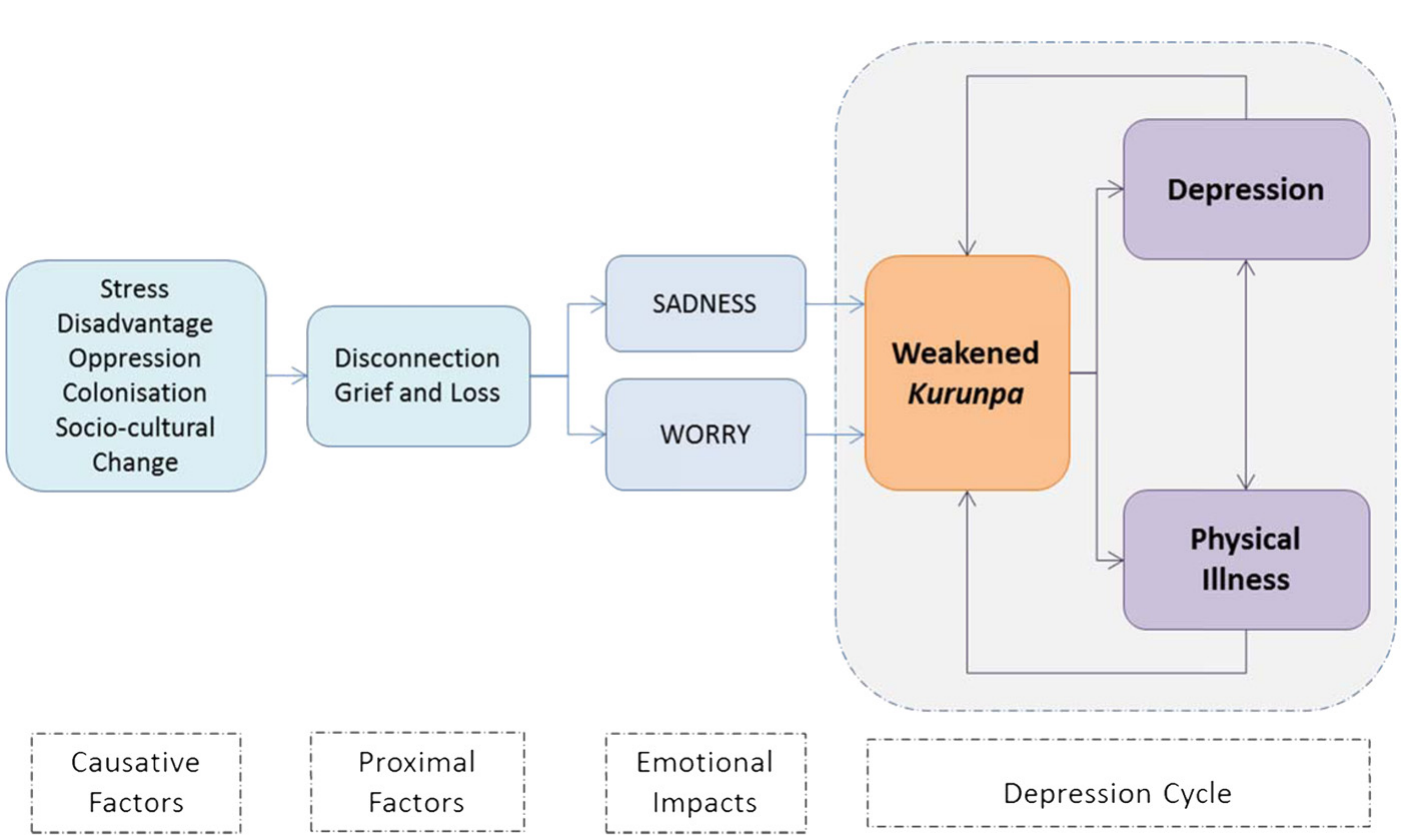
Understanding Depression in Aboriginal Men.

In our study, a weakened, displaced or misaligned spirit [*Kurunpa*] was the primary explanation and expression of depression in Aboriginal men. Figure 1 is a representation of the conditions whose interaction, we theorise, manifests as depression in Aboriginal men. In this model, depression was ‘caused’ by the pervasive and cumulative impact of chronic stress, the experience of socioeconomic disadvantage, and the down-stream impact of colonisation, through the lived experience of oppression and rapid and severe socio-cultural change [*Causative Factors*]. More proximally, the consequence of socio-cultural change and marginalisation was understood and explained as disconnection. This was experienced as forced and painful separation from the fundamental essential elements of Aboriginal life and Aboriginal ways of being. From family, the law, from their land, and a forced segregation from their capacity to give and receive care to the people and landscape held within their construction of family. Separation from all they held dear had significant negative emotional, spiritual and physical consequences. Most critically, these negative influences had significant *emotional impacts*, most commonly experienced and understood as great sadness, and repetitive, intrusive worry. These factors had a direct and painful influence on the spirit, *Kurunpa*, serving to weaken, misplace, or injure it. As a consequence, a weakened spirit had recognizable and consistent impacts on emotional well-being, directly leading to depression, but also contributed to the development of physical illness. Physical *and* emotional illness, was not only a consequence of a weakened spirit, but served to further negatively influence the spirit itself [*Depression Cycle*].

Our emerging theory of depression in Aboriginal men extends the existing literature in the differentiation of experiences, trajectories and explanations of cumulative stressors that contributed to depressive symptoms across ages. The stories and context of depressive symptoms in younger males were framed within the realms of the social world, with significant and repetitive social chaos contributing to depression. There were heightened pressures felt by young men, including the ‘fit’ between themselves and the dominant culture of mainstream Australia, the pressure to maintain an increasingly fragmented ‘Law’ through cultural preservation and reinvigoration, the disconnection they felt within their families and communities, and as a consequence of the trans-generational impact of forced separation. These increasingly heavy and cumulative burdens manifest as a sense of inner turmoil and questioning of self, and of feelings of disconnectedness from all the things of importance within their lives. Escalating frustration was common, particularly against a backdrop of a sense of powerlessness to make significant changes within their lives. Many young men turned to substance misuse as a means of suppressing or numbing their inner turmoil, feelings which were rarely discussed openly. However, these strategies simply delayed the inevitable buildup of anger, which subsequently spilled over into self-destructive behaviour.

In older Aboriginal men, narratives were melancholic reflections of the cumulative impact of loss and grief throughout their lives. This loss was most particular for friends and family, however, the narratives also focused on mourning for the loss of the ways of their grandfathers. Positioned beyond some romantic notion of a traditional culture, their grief was framed by the lack of direction and clearly defined roles for the younger generations as they struggled to cope with an ever changing world.

Whilst the celebration and re-invigoration of cultural life among these older participants were seen as pivotal to the well-being of men, it was the lack of purpose and guidance for the younger generations that was most acutely observed. Without Law and culture, younger Aboriginal men were being ‘sentenced’ to a future without clarity in relation to an Aboriginal identity. This severance from the foundations on which Aboriginal constructions of well-being were built - the Law [*Tjukurpa*], Family [*Walytja*], the Land [*Ngurra]*, and the sense and obligations to care for and remain connected to the social, physical and emotional world around them [*Kanyini*] - was for these participants the primary explanation for the increasing burden of emotional distress in Aboriginal men. Fundamentally, senior Aboriginal men bore witness to the world they had known now disintegrating around them, and this contributed to significant sorrow.

These feelings and subsequent emotional consequences are central to understanding the social nature of depression within Aboriginal men [10]. Socio-cultural change, in many respects imposed on Aboriginal men by external forces, has been compressed into a relatively short period of time, and has had devastating impacts on clarity in, and opportunities to self-define and enact, the roles and identity of Aboriginal men within contemporary society [49].

In contrast to the very concrete stories of cumulative stress, distress and despair apparent among younger and senior Aboriginal men, the understandings shared by *Ngangkari Tjuta* were more metaphysical. *Ngangkari* were the primary point of contact for Aboriginal men with emotional distress in remote Aboriginal communities. *Ngangkari* explained their continuing role within contemporary Aboriginal life as ‘conduits’ or ‘guides’ for community members to link and understand both the physical and spiritual worlds, and their task of transiting between the spiritual (thus emotional) and physical expressions of sadness, worry and depression. *Ngangkari Tjuta* discussed their connectedness to *Kurunpa*, which they were able to visualise, diagnose, manipulate, replace and heal. *Kurunpa* is the foundation of vitality and is critical to the physical, emotional and spiritual well-being of Aboriginal men. It exists in physical, emotional and spiritual form, which can be injured, manipulated, moved, lost, felt, seen, found, and replaced. *Kurunpa* goes beyond metaphor; it is not only a feeling, or a means of expressing distress: it is the vessel of life force itself.

Depression was seen as both a contributor to and consequence of displacement, disconnectedness, distortion or injury of the spirit. All visible, cognitive and emotional manifestations of depression were a direct consequence of the impact of worry and sadness on the health and vitality of an individual’s spirit. Importantly, many of the identified symptoms of depression were easily recognized and their cause understood by *Ngangkari* based on their traditional knowledge and practice. Expression of depression within the model of *Kurunpa*, placed distress within the direct healing realm of *Ngangkari*, whose primary repertoire involves the manipulation, centering, and diagnosis of spirit. This not only creates the context for understanding depression within Central Australian Aboriginal men, but highlights the critical role of *Ngangkari* in the preservation of mental health and healing of illness.

Consistent with a broader literature on mental illness among Indigenous Australians [7,10,24,50], our study documented a holistic construction of physical, emotional and spiritual well-being. Categorisation of the body, emotions and spirit as inherently separate dimensions of self, proved problematic, and may explain the lack of a consistent somatic patterning to depressive symptoms in this population. Given that the expression of self (as Aboriginal) is bound to the very essence of life (*Kurunpa*), which is the primary target for negative experience, then a *Kurunpa* which is ill or injured (through whatever means or mechanisms), manifests as undifferentiated emotional, mental, cognitive or physical symptoms. In essence, the conscious classification of separate parts of the whole experience of depression is meaningless within a culture which neither knows nor expresses a separation.

Of particular note was that the descriptions of worry [rumination] were not focused on ‘the self’ in any way, nor were they associated with self-perceived deficit. Importantly, rumination focused on concern for others. As discussed within Myers' ethnography of the Pintupi [37], self, particularly personal autonomy, is well recognised and valued within Aboriginal life. However, the essence of self is *defined by* and *grounded within* the well-being of others. To be healthy, all things of importance to Aboriginal people must also be healthy [51,52]. To be Aboriginal, is to care for others [37,53,54] and thus self *is* a function of others. This concept may also help explain the lack of articulation of hopelessness within these narratives. Without exception, the focus for Aboriginal men’s aspirations was never for self, but always for others. These concerns formed the primary themes of the recurrent worry within Aboriginal men’s lives. Given that they were largely outside of men’s control, they did not contribute to self-reproach, self-doubt and hopelessness. Hopelessness, as framed within self-deficit, is replaced by the intrusion and injury caused by externalities, from the social contributors to depression and illness of spirit. This then contributes to the sense of loss and harm from without, manifesting as grief, sadness and worry, rather than self-deficit and reproach.

Profound disadvantage and cumulative stress within Aboriginal people’s lives may also render concern for and conceptualisation of the future as a luxury few could afford. One could also argue that a lack of future perspective is likely to inhibit the expression of hopelessness within this group. If people do not conceptualise the future, then one is unlikely to *lose* hope for their own aspirations in years to come. Yet to infer lack of consideration for the future as ubiquitous among Aboriginal men is simplistic and flawed. Following the final interviews, our analysis had yet to adequately understand the seeming invisibility of hopelessness within participant’s narratives. It was not until we continued our key informant conversations that a senior elder explained:

"*There is no word for future in [our] language…it’s because we never face the future, we can’t see it, we don’t know it. We walk towards the future backwards…looking to the past. The past we can see and we know. Our past is what helps us survive anything that will come.*"

"Senior Aboriginal Man, Remote Community."

Clearly, further exploring the expression and conceptualisation of hopelessness and related constructs (or lack thereof) is essential to unpacking the mental health issues facing Indigenous communities.

In terms of clinical relevance, this work reinforces how unlikely it is that Aboriginal men will simply present to their primary care providers to discuss the intricacies and contributors to emotional distress. Detailed and explicitly developed methodologies were required to commence discussions about feelings. For many participants, involvement in this research was the first time they had ever discussed emotions, frustrations and contributors to their anger and sadness. Whilst it would be easy to claim this as a culturally specific issue, existing evidence suggests that the expression of depression and emotion in men of many cultural groups is muted [55]. Importantly, analysis of these interviews suggests that there are key windows of opportunity to commence discussions with Aboriginal men about depression and emotion. Worry assumes critical relevance, and the nature of these worries may assist in not only identifying depression in Aboriginal men, but in identifying therapeutic targets and opportunities [56,57]. Questions about substance misuse (particularly recent changes in substance use), alongside assessment of suicidality, may also serve as key signposts. Finally, one has to question the most appropriate therapeutic options for Aboriginal men experiencing depression. Whilst much of the available evidence base supports the role of cognitive behavioural therapy [58,59], given the nature and contributors to depression in Aboriginal men identified in this study, one must ask whether Aboriginal men’s cognitions remain distorted or flawed, and thus amenable to reorientation, or whether or not they could be seen as realistic appraisals of the social chaos, grief and questioning of identity that faces them on a recurrent basis.

## Conclusion

These accounts of depressive symptoms and their construction among Aboriginal men represent a unique exploration of emotion, language, Indigenous philosophy and models of illness and health. Depression and depressive symptomatology clearly exists in Aboriginal men, even in diverse remote community settings. Emotions were framed and understood within the construction of spirit, *Kurunpa*, the life force and essence of Aboriginal life which was vulnerable to the impact of recurrent trauma, grief and loss, social chaos, sorrow and despair. Detailed understanding of the causes and expression of depressive symptoms and emotions among Aboriginal people is essential to improving the well-being of Australia’s most vulnerable people.

## Competing interest

All authors with the exception of U.S. have completed the Unified Competing Interest form at http://www.icmje.org/coi_disclosure.pdf (available on request from the corresponding author) and declare that the authors have received no support from any companies for the submitted work; have no relationships with any company that might have an interest in the submitted work in the previous 3 years; and have no financial or non-financial interests themselves or among family that may be relevant to the submitted work. The family of U.S. have completed the Unified Competing Interest form on his behalf.

## Authors’ contributions

AB contributed to study conception and design, data collection, analysis and interpretation of the data and drafting of the manuscript. US contributed to the study design, data collection, data interpretation and translation. WB assisted in data collection and review of the manuscript. BR assisted in data analysis and interpretation and drafting review of the manuscript. KR contributed to study design, data analysis and drafting of the manuscript. KO’D contributed to study conception and design, interpretation of the data and drafting of the manuscript. All authors read and approved the final manuscript.

## Pre-publication history

The pre-publication history for this paper can be accessed here:

http://www.biomedcentral.com/1471-244X/12/97/prepub

## Supplementary Material

Additional file 1 ** Appendix 1.**Introductory Vignette – *Kunmanara's* Story. This is the introductory vignette utilised at the commencement of the participant interviews to ground the interview schedule. Click here for file

Additional file 2 ** Appendix 2.**Interview Schedule. The semi-structured interview schedule utilised throughout the interview process Click here for file

Additional file 3 ** Appendix 3.**Depression Symptom Endorsement Inventory. Iteratively developed symptom checklist for participants to consider/endorse. Click here for file
