# Supplementary material for: Exploring the expression of depression and distress in aboriginal men in central Australia: a qualitative study
Source: BMC Psychiatry. 2012 Aug 1;12:97. doi: 10.1186/1471-244X-12-97 (PMC3441213; doi:10.1186/1471-244X-12-97)
Supplement: Additional file 1 — Appendix 1.Introductory Vignette – Kunmanara's Story. This is the introductory vignette utilised at the commencement of the participant interviews to ground the interview schedule. [file 1471-244X-12-97-S1.docx]

**Appendix 1:**

**Introductory Vignette – *Kunmanara's* Story**

Kunmanara is a 52 year-old man. He comes from a large community, with a number of homelands scattered around it. When he was younger, he used to work as a stockman, and was considered a very good horseman. He moved back to his community from the station when he got married. Since then he sometimes does work at the community council. He finds this very frustrating because he always has to work with new community advisors, and trouble in the community never seems to get any better.

Kunmanara lives with his wife and three children. His eldest son spends most of his time in Alice Springs drinking and is often in trouble. He only ever really visits when he needs some money. Kunmanara’s daughter-in-law used to live here with her three children, but left for her mother’s community when Kunmanara’s son started drinking a lot. This caused Kunmanara lots of worry and sadness. Recently, however, one grandson returned to live with him and his wife which was a cause of great happiness for Kunmanara. He is a good young footballer, a cause for great pride for Kunmanara.

Kunmanara used to drink grog when he was younger, but stopped when he had his first child. He thinks a lot about taking the family to their homelands, but his son took his car and it got damaged in Alice Springs, and he hasn’t been able to save any money for repairs. His mates have noticed that he is not as interested in going hunting as he used to be.

Kunmanara has had trouble with diabetes in the last few years, and is on medicine for his sugar. He occasionally suffers from pain in his knee, caused by a fall from a horse many years ago, but is pretty fit and healthy. In the last few months, Kunmanara has noticed that he is unable to sleep as well as he used to, waking early in the morning. He is not as full of energy as usual, and he doesn’t eat much and gets occasional headaches.

He goes to the clinic to get a check up and the male nurse and Aboriginal Health Worker sits down for a talk. The nurse asks him how he has been and Kunmanara says that he has been thinking a lot about the old days and what he misses most about them. He tells the nurse that he been thinking a lot about his kids and grandkids, and would love to take them to the family homeland.

The nurse gives him a well men’s check-up, and then asks Kunmanara about how he is feeling. He asks him whether he is feeling sad, worried, how he is sleeping, whether he has any aches and pains, or if he feels depressed.

Kunmanara turns to the Health worker and asks in language “what does he mean depressed”?
