# Supplementary material for: Exploring the expression of depression and distress in aboriginal men in central Australia: a qualitative study
Source: BMC Psychiatry. 2012 Aug 1;12:97. doi: 10.1186/1471-244X-12-97 (PMC3441213; doi:10.1186/1471-244X-12-97)
Supplement: Additional file 2 — Appendix 2.Interview Schedule. The semi-structured interview schedule utilised throughout the interview process [file 1471-244X-12-97-S2.docx]

**Appendix 2:**

**Interview Schedule**

- Have you seen someone experiencing troubles like Kunmanara [trouble sleeping, no energy, less appetite, headaches, aches and pains, loss of interest in usual activities, worry]?
- Are there words for or ways of describing people feeling like Kunmanara?
- Are there other ways of acting, thinking or feeling that would be a sign of these troubles?
- What are the sicknesses that can be wrong with peoples’ mind?
- What are the sicknesses that can be wrong with peoples’ spirit?
- How could we find out if someone is feeling these ways?
- Do you understand what the nurse meant when he asked Kunmanara about ‘depression’?
- How would family talk about (describe) someone who is feeling depressed?
- How can you tell if someone is depressed [*or related terms*]?
- What are the causes of these problems?
- Do [*types of people*] feel like this? (eg. Young males, petrol sniffers, dope smokers, people who have been to prison, drinkers, old men, people who have attempted to kill themselves, etc.).
- What is the difference between people feeling depressed and people who are *ramarama* [crazy]?
- In the olden days, where there people who suffered from Depression?

We are now going to list symptoms that doctors and mental health workers often use to describe or ask people about depression. Can you tell us if *Anangu* men with depression [*or related terms*] also have these symptoms?
