# Supplementary material for: Exploring the expression of depression and distress in aboriginal men in central Australia: a qualitative study
Source: BMC Psychiatry. 2012 Aug 1;12:97. doi: 10.1186/1471-244X-12-97 (PMC3441213; doi:10.1186/1471-244X-12-97)
Supplement: Additional file 3 — Appendix 3.Depression Symptom Endorsement Inventory. Iteratively developed symptom checklist for participants to consider/endorse. [file 1471-244X-12-97-S3.docx]

| **Depression Symptom Endorsement Inventory** | |
| --- | --- |
| ***List of commonly used terms to describe depressive mood/associated symptoms*** | |
| **Mood/Feeling**  Excessive Sadness / Grief  Depressed  Hopelessness (‘without hope’)  People feel ‘empty’  ‘Down in the Dumps’ / ‘Feeling Blue’  Loss of enjoyment/interest in activities that usually make that person happy  People become irritable (‘cranky’) |  |
| **Physical Symptoms**  Change in appetite or weight (usually loss)  Change in sleeping patterns  (usually trouble getting to sleep and waking up very early in the morning)  Tiredness/loss of energy/feel ‘slowed down’  Slowing of speech, movements and thinking  Muscles aches and pains  Headaches  Upset stomach/ GIT disturbances |  |
| **Cognition / Thinking**  Unusual self-reproach (feels bad about themselves / feels that they are ‘no good’) |  |
| Excessive Guilt  Poor concentration (gets tired when thinking, cannot remember things)  Indecisive (cannot make a decision)  Thinks about or attempts suicide  Rumination |  |
| **Other Symptoms**  Anxiousness  Fear  Loneliness  Longing for family or country (Watjilpa)  Weak Spirit (Kurunpa)  Withdrawal  Self-harm  Despair  Disconnection from family/culture/others  Violence, rage  Somatic complaints [head, throat, stomach, muscles/joints, pain] | |

**Appendix 3**:
